# The Effect of ShenQi FuZheng Injection in Combination with Chemotherapy versus Chemotherapy Alone on the Improvement of Efficacy and Immune Function in Patients with Advanced Non-Small Cell Lung Cancer: A Meta-Analysis

**DOI:** 10.1371/journal.pone.0152270

**Published:** 2016-03-25

**Authors:** Cao dedong, Xu huilin, He Anbing, Xu Ximing, Ge wei

**Affiliations:** 1 Department of oncology, RenMin Hospital of Wuhan University, Wuhan, China; 2 Department of oncology, The fifth hospital of Wuhan, Wuhan, China; Stavanger University Hospital, NORWAY

## Abstract

**Objective:**

To evaluate the effect of ShenQi FuZheng Injection (SFI) on cellular immunity and clinical efficacy in patients with advanced non small cell lung cancer(NSCLC) when combined with chemotherapy.

**Methods:**

Electronic databases including EMBASE, PUBMED, the conference proceedings of the American Society of Clinical Oncology (ASCO), Cochrane, Chinese National Knowledge Infrastructure (CNKI), and Chinese Biological Medical disc(CBM) were searched, until July, 2015. The randomized controlled clinical studies reporting results of efficacy and immune function were collected according to the inclusion criteria. Cochrane handbook 5.1.0 was applied to assess the quality of included trials and Revman 5 software was used for data analysis.

**Results:**

Fifteen studies including 1006 cases of advanced NSCLC were included based on the inclusion criteria. The results of meta-analysis showed that there were significant differences in percentages of CD_3_^+^ cells (SMD = 13.48; 95%CI: 8.11–18.85; p<0.01), CD_4_^+^ cells (SMD = 10.78; 95%CI, 6.38–15.18; *p*<0.01), NK [WMD = 8.59, 95% CI(3.97, 13.21), p = 0.003], and ratio of CD_4_^+^/ CD_8_^+^ (SMD = 0.32; 95%: 0.28–0.36; *p*<0.01) between SFI combination group and control group, whereas the difference was not significant in CD_8_^+^ (SMD = -1.44; 95%CI, -4.53–1.65; *p* = 0.36). Funnel plot, Begg's rank correlation test and Egger's linear regression analysis indicated that there was significant publication bias across studies.

**Conclusion:**

SFI is effective to improve the efficacy of chemotherpay and function of cellular immunity in NSCLC patients, however, high quality RCTs are needed to further confirm the findings.

## Introduction

Lung cancer is the leading cause of cancer-related mortality worldwide, and it accounts for approximately 1.4 million deaths every year[1]. In China, the incidence of lung cancer was significantly increased during the past 20 years. About 600,000 patients lost their lives because of lung cancer, according to the released data from the National Office on Tumor Cure and Prevention, and the death caused by lung cancer may be even higher in 2025, estimated by The World Health Organization[2].

It is estimated that approximately 85% of lung cancer are non-small cell lung cancer(NSCLC). Importantly, most of these NSCLC patients are inappropriate to receive resection as they reach advanced stages at the time of diagnosis. Platinum based chemotherapy is usually preferred for lung cancer patients with advanced stages, and these regimen indeed achieved favorable outcomes[3]. However, besides its curative effect, hematal and gastrointestinal toxicity of regimen containing platinum should also be noticed, which may severely affect the quality of life, immune function and efficacy of these patients[4]. The immune functions of cancer patients are relatively hampered[5]. In addition, in recent years, the immune therapy of tumors has been extensively focused, and shows powerful effective results[6]. So, how to improve the immune function, quality of life, and reduce toxicity during platinum based chemotherapy is now becoming extremely urgent.

Traditional Chinese medicines (TCMs) have been widely used in China for treating cancers[7]. Shenqi Fuzheng Injection (SFI) is extracted from two different Chinese herbs, Radix codonopsis and Radix astragali, at an even ratio[8]. SFI was approved by China's State Food and Drug Administration years ago, and generally used to modify the immune function of patients with malignant tumors, such as NSCLC, gastric cancer, and liver cancer, etc[9]. A number of published studies have proved that SFI showed synergetic anti-tumor effects when combined with chemotherapy by improving objective response rate, increasing the Karnofsky score (KPS), enhancing the immune functions, and reducing the occurrence of adverse events during chemotherapy[7, 9–11]. Indeed, a previous research showed that proper duration of treatment with SFI resulted in accelerating recovery of immunosuppression in cytotoxic agent-treated mice[8]. A systematic meta-analysis including 13 randomized controlled trials(RCTs) was performed to evaluate the curative effect of SFI in patients with advanced gastric cancer[9]. The results exhibited that the combination of chemotherapy with SFI achieved a series of effective outcomes including improvement of quality of life, complete response(CR) and partial response(PR), and reduction of adverse events such as oral mucositis, leucopenia, nausea and vomiting.

Thus, we conducted this meta-analysis with the aim of assessing the clinical efficacy and immune-enhancement of SFI combined with chemotherapy in the treatment of advanced NSCLC.

## Methods

### Inclusion and exclusion criteria

(1) Patients: NSCLC confirmed by cytology or pathology, with advanced stages. (2) Type of Study: Randomized controlled clinical trial (RCT). (3) Intervention: SFI combined with platinum-containing chemotherapy versus platinum-containing chemotherapy alone. (4) Outcomes: Relevant indicators of cellular immune function, including percentages of total T lymphocytes (CD_3_^+^), helper T lymphocytes (CD_4_^+^), cytotoxic T lymphocytes (CD_8_^+^), CD_4_^+^/ CD_8_^+^, natural killer cells (NK), concentration of IL-2, TNF-alpha, quality of life, adverse events, complete response(CR), and partial response(PR).

### Search strategy

Online databases including EMBASE, PUBMED, the conference proceedings of the American Society of Clinical Oncology (ASCO), Cochrane, Chinese National Knowledge Infrastructure (CNKI), and Chinese Biological Medical disc(CBM) were selected and searched for identification of eligible studies by Cao dedong and Xu huilin. Studies aiming to compare the clinical efficacy and immune-enhancement of SFI combined with chemotherapy versus chemotherapy alone were regarded as potential sources of this meta-analysis and included after systematically review. The languages of included studies were limited to Chinese and English, with the restriction of human trials. The key terms used in this search were as follows: "lung cancer/NSCLC/non-small cell lung cancer", "Shenqifuzheng", "SFI", and "chemotherapy".

### Data extraction and quality assessment

The required data from each included studies using previously designed tables were extracted by Cao dedong and He anbing, independently. Any problems or disagreements were solved by discussion. All included studies were reviewed and approved by at least two reviewers. General information of the eligible studies including name of the first author, number of participants in treatment group and control group, sex, age, chemotherapy regimen, and details of SFI treatment were selected from each article. Specific data such as clinical efficacy, immune function, safety and quality of life were also carefully extracted by Cao dedong and He anbing. The individual quality of included studies were also assessed based on the standards of evaluation of quality in Cochrane Handbook. Briefly, the main questions about quality were randomization, concealment of allocation, selective reporting of results, and other sources of bias.

### Statistical analysis

The software RevMan5.3(Cochrane Collaboration) was used to perform the meta-analysis. The Z-test analysis and I^2^ test were introduced to evaluate the overall heterogeneity of included studies. The estimated outcomes across included trials were calculated with the random effect model if I^2^>50%, otherwise the fixed effect model was used[12]. For dichotomous data, the pooled odd ratio(OR) were calculated with the 95% confidence interval (CI). For continuous data, the weighted mean differences(WMD) were calculated with the 95%CI, and standardized WMD(SMD) were also calculated for every trial involved in the meta-analysis[13]. The main end points of this study were OR of objective response rates and QOL improvement, SMD of immune function improvement. For detection of publication bias, funnel plot based on trials with data on response, immune function was performed and Egger’s test was also introduced.

## Results

### Search results

**Fig 1:**
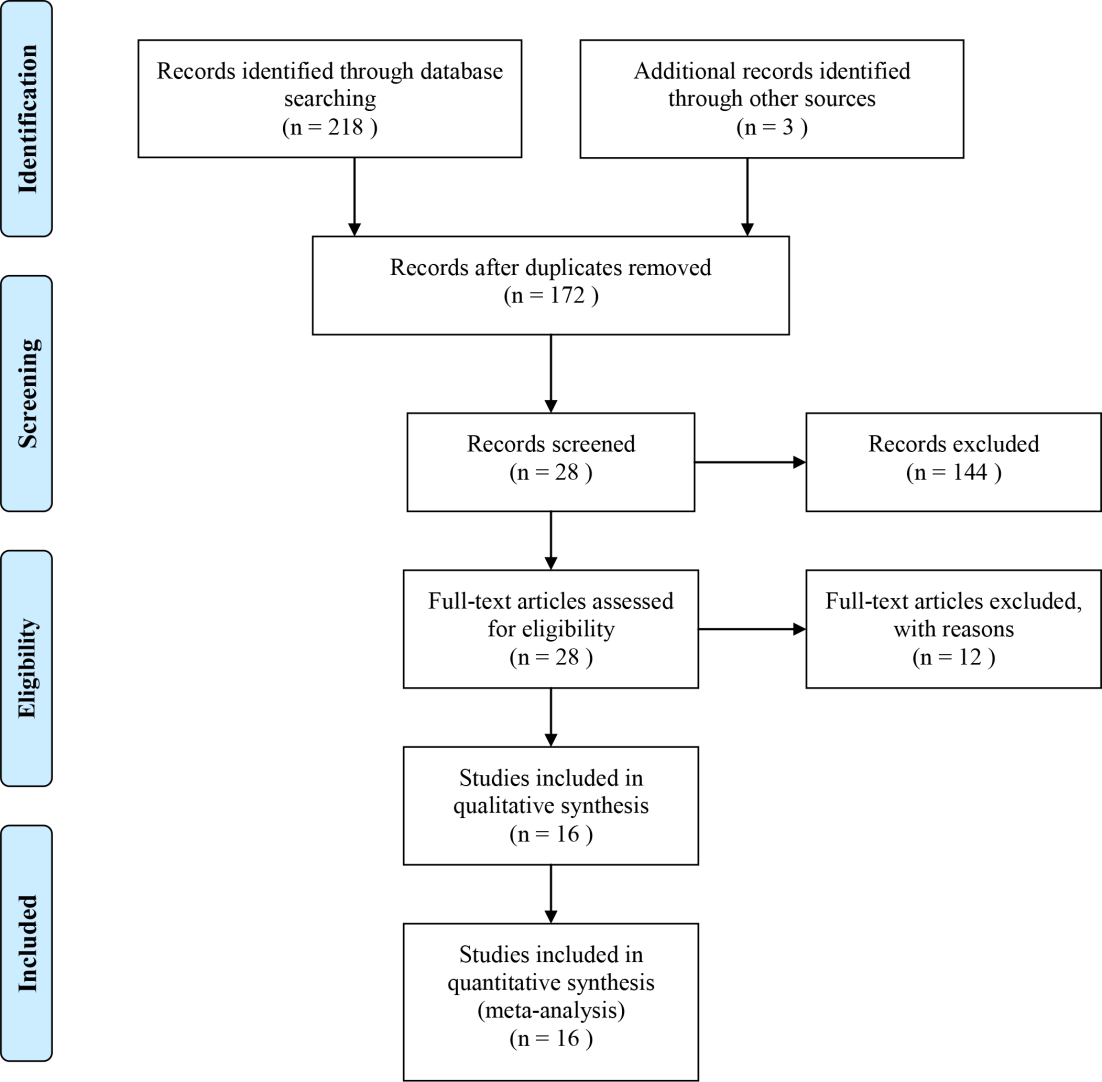
Flow Diagram of Searching for Eligible Studies.

The process of selection of the eligible studies was illustrated in Fig 1 in the form of PRISMA flow diagram. Briefly, the systematic search was conducted on July 1th, 2015, and 218 citations were found through online databases when the primary search was over. Another 3 records were included by reviewing other sources. A total of 172 articles were left and entered next step of screening after removal of duplications. After further review, 15 publications were regarded as eligible for the analysis. The reasons for exclusion of the other studies were review article, studies without reporting immune function, one arm study, and animal study(S1 Table). Finally, a total of 15 trials[10, 11, 14–26] were included and the data extracted from these studies were used in the qualitative analysis.

### Baseline characteristics of identified studies

Table 1 presents a summary of the baseline characteristics of included studies. All these studies were randomized controlled trials and conducted in China. The dates of publication were between 2004 and 2015. The stages of the reported patients were advanced stages. The duration of the SFI treatment ranged from 10 days to 30 days. Three of the studies[14, 15, 23] used SFI plus GP regimen, eight[11, 15, 16, 20, 22, 24–26] used SFI plus TP regimen, two[17, 19] used SFI plus NP regimen, one[21] study used SFI plus EP regimen. Nine[10, 14, 15, 17, 19, 21–23, 25] of the trials reported outcomes of tumor response, fourteen[10, 11, 14–21, 23–26] showed outcomes of immune function, and six[14, 17, 19, 21, 23, 25] reported numbers of patients achieved improvement of quality of life.

**Table 1:** Baseline characteristics of included studies. Abbreviation: KPS, karnofsky; SFI, shenqi fuzheng injection; T, treatment; C, control; Y, year; GP, gemcitabine + platinum; TP, taxol + platinum; NP, navelbine + platinum; EP, etoposide + platinum; BST, best support care; NK, natural killer cells.

Studies	N(T/C)	Sex(M/F)	Age(Y)		KPS	Stage	Intervention		Immune function
			T	C			T	C	
Ding CJ, 2012	35/35	42/28	38~70	40~70	≥70	ⅢB~Ⅳ	GP/TP+SFI (SFI 250mL/d,d1-d10)	GP/TP	CR, PR, ORR, CD_3_^+^, CD_4_^+^, CD_8_^+^,CD_4_^+^/ CD_8_^+^, NK(%)
Zhou T, 2009	35/35	43/27	34~70		>60	ⅢB~Ⅳ	GP+SFI (SFI250mL/d,d1-d10)	GP	CD_4_^+^/ CD_8_^+^, NK(%)
Sun YY, 2007	34/28	41/21	44~69	41~71	>60	ⅢA~Ⅳ	TP+SFI (SFI250mL/d)	TP	CD_3_^+^, CD_4_^+^, CD_8_^+^,CD_4_^+^/ CD_8_^+^, NK(%)
Geng L, 2004	25/15	25/15	25~64	25~68	≥70	Ⅲ~Ⅳ	NP+SFI (SFI250mL/d)	NP	CD_3_^+^, CD_4_^+^, CD_8_^+^,CD_4_^+^/ CD_8_^+^, NK(%)
Lu QG, 2011	33/29	35/27	65~81	67~81	>60	Ⅲ~Ⅳ	EP+SFI (SFI250mL/d)	EP	CD_4_^+^, CD_8_^+^,CD_4_^+^/ CD_8_^+^, NK(%)
Jiang Y, 2005	35/32	53/14	28~74	27~73	>60	Ⅲ~Ⅳ	TP+SFI (SFI250mL/d)	TP	CD_3_^+^, CD_4_^+^, CD_8_^+^,CD_4_^+^/ CD_8_^+^, NK(%), IL-2,LAK
Zhang LL, 2009	30/30	32/28	45~70		≥70	Ⅲ~Ⅳ	NP+SFI (SFI250mL/d)	NP	CD_3_^+^, CD_4_^+^, CD_8_^+^,CD_4_^+^/ CD_8_^+^
Niu LJ, 2015	17/17	24/10	45~76		>60	Ⅲ~Ⅳ	SFI (SFI250mL/d,d1-d10)	BST	CD_4_^+^, CD_8_^+^,CD_4_^+^/ CD_8_^+^, NK(%)
Qiao SL, 2012	30/30	36/24	61.2		>60	Ⅲ~Ⅳ	TP+SFI (SFI250mL/d,d1-d10)	TP	CR, PR, ORR
Lin CH, 2014	32/30	40/22	44~70	41~69	>60	Ⅲ~Ⅳ	GP+SFI (SFI250mL/d,d1-d10)	GP	CR, PR, ORR, CD_3_^+^, CD_4_^+^, CD_8_^+^,CD_4_^+^/ CD_8_^+^
Ren JS, 2015	42/42	49/35	52~73	53~72	>60	Ⅲ~Ⅳ	PP+SFI (SFI250mL/d,d1-d10)	PP	CR, PR, ORR, CD_3_^+^, CD_4_^+^, CD_8_^+^,CD_4_^+^/ CD_8_^+^
Liu YF, 2011	50/50	51/49	43~72		>60	Ⅲ~Ⅳ	TP+SFI (SFI250mL/d,d1-d10)	TP	CD_3_^+^, CD_4_^+^, CD_8_^+^,CD_4_^+^/ CD_8_^+^, NK(%), IL-2,
Wang J, 2013	28/15	27/16	50~73		>60	Ⅲ~Ⅳ	TP+SFI (SFI250mL/d,d1-d10)	TP	CD_3_^+^, CD_4_^+^, CD_8_^+^,CD_4_^+^/ CD_8_^+^, NK(%)
Ao M, 2012	30/25	NA	35~74		>70	Ⅲ~Ⅳ	TP+SFI (SFI250mL/d,d1-d10)	TP	CD_3_^+^, CD_4_^+^, CD_8_^+^,CD_4_^+^/ CD_8_^+^, NK(%)
Ren L, 2014	65/72	89/48	66.5 ± 15.3	65.9 ± 14.7	>60	Ⅲ~Ⅳ	TP+SFI (SFI250mL/d,d1-d10)	TP	CD_3_^+^, CD_4_^+^, CD_8_^+^,CD_4_^+^/ CD_8_^+^, NK(%)

The overall quality of the included studies were mild due to the following reasons: lack of blinding, incomplete reporting and allocation bias. The detailed information of quality assessment is listed in Table 2.

**Table 2:** Methodological quality evaluation of included studies. Abbreviation: Y, yes; NA, not available; NR, not reported.

Studies	Random	Allocation	Blinding	Incomplete outcome data	Selective reporting	Other bias
Ding CJ, 2012	Y	NA	N	N	N	NR
Zhou T, 2009	Y	NA	N	N	N	NR
Sun YY, 2007	Y	NA	N	N	N	NR
Geng L, 2004	Y	NA	N	N	N	NR
Lu QG, 2011	Y	NA	N	N	N	NR
Jiang Y, 2005	Y	NA	N	N	N	NR
Zhang LL, 2009	Y	NA	N	N	N	NR
Niu LJ, 2015	Y	NA	N	N	N	NR
Qiao SL, 2012	Y	NA	N	N	N	NR
Lin CH, 2014	Y	NA	N	N	N	NR
Ren JS, 2015	Y	NA	N	N	N	NR
Liu YF, 2011	Y	NA	N	N	N	NR
Wang J, 2013	Y	NA	N	N	N	NR
Ao M, 2012	Y	NA	N	N	N	NR
Ren L, 2014	Y	NA	N	N	N	NR

### Efficacy of SFI combined with chemotherapy

**Fig 2:**
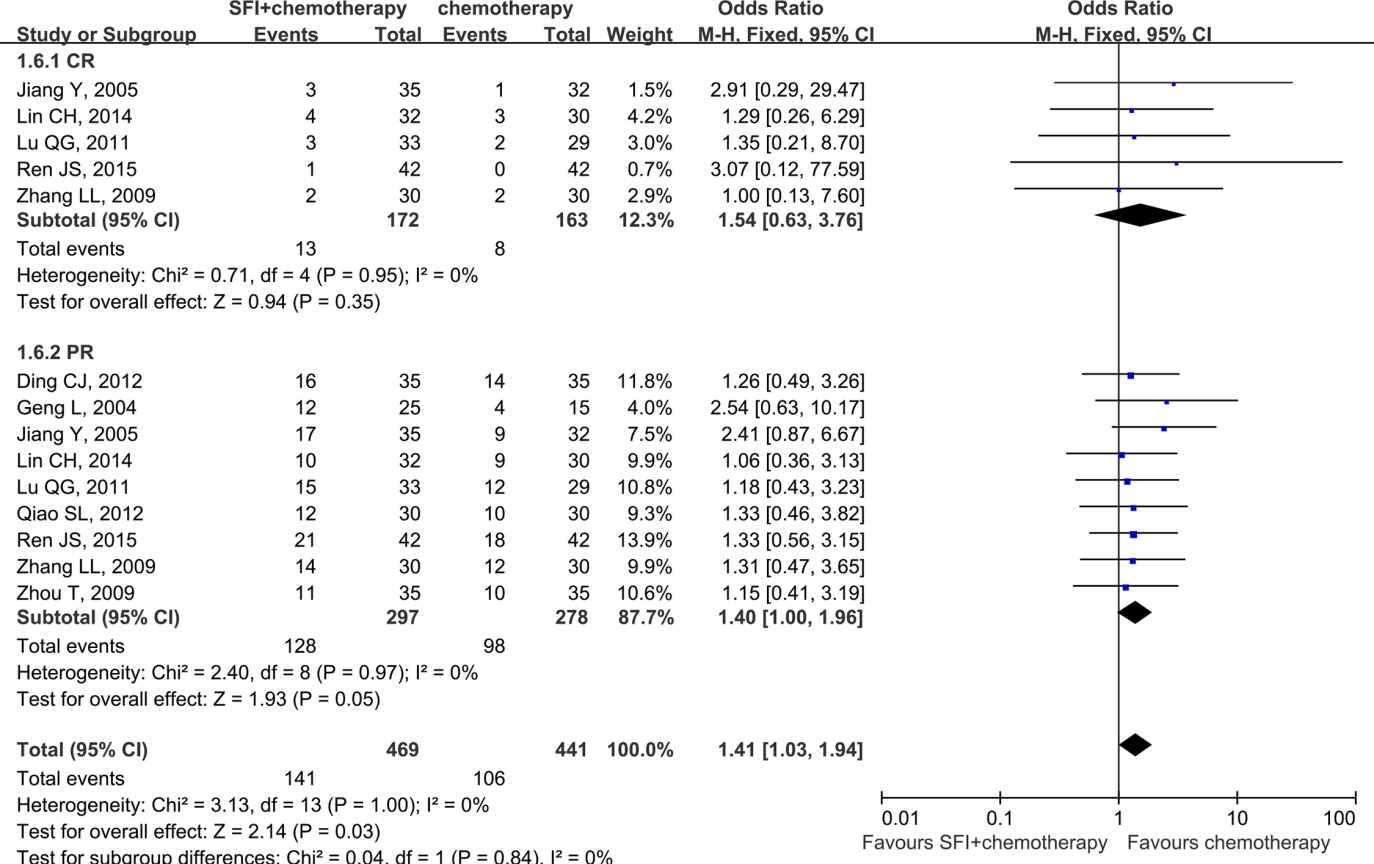
Pooled results of efficacy of SFI combined with chemotherapy for advanced NSCLC patients.

The outcomes of tumor response were reported in nine trials[10, 14, 15, 17, 19, 21–23, 25], which included 575 NSCLC patients. In the SFI combined with chemotherapy group, 141 of 297(47.5%) patients reached CR or PR, while 106 of 278(38.1%) subjects achieved CR or PR in the chemotherapy alone group. These studies also reported the disease control rate and stable disease rate. The value of I^2^ was less than 50%, indicated that the heterogeneity across different trials was not significant(*P>*0.05), and the fixed effect model was applied in this pooled analysis. As shown in Fig 2, the result of meta-analysis demonstrated a favorable OR for SFI treatment(OR = 1.47, 95%CI:1.05–2.06; *p* = 0.02). However, in the subgroup analysis, there were no significant differences with regards to either CR or PR between combination group and control group. The pooled OR for CR was 1.54 with 95%CI ranged from 0.63 to 3.76(*p* = 0.35), and the integrated OR for PR was 1.40(95%:1.00–1.96; *p* = 0.05). These findings indicated that SFI combined with chemotherapy had better ORR than chemotherapy alone.

### Effect of SFI on immune function

**Fig 3:**
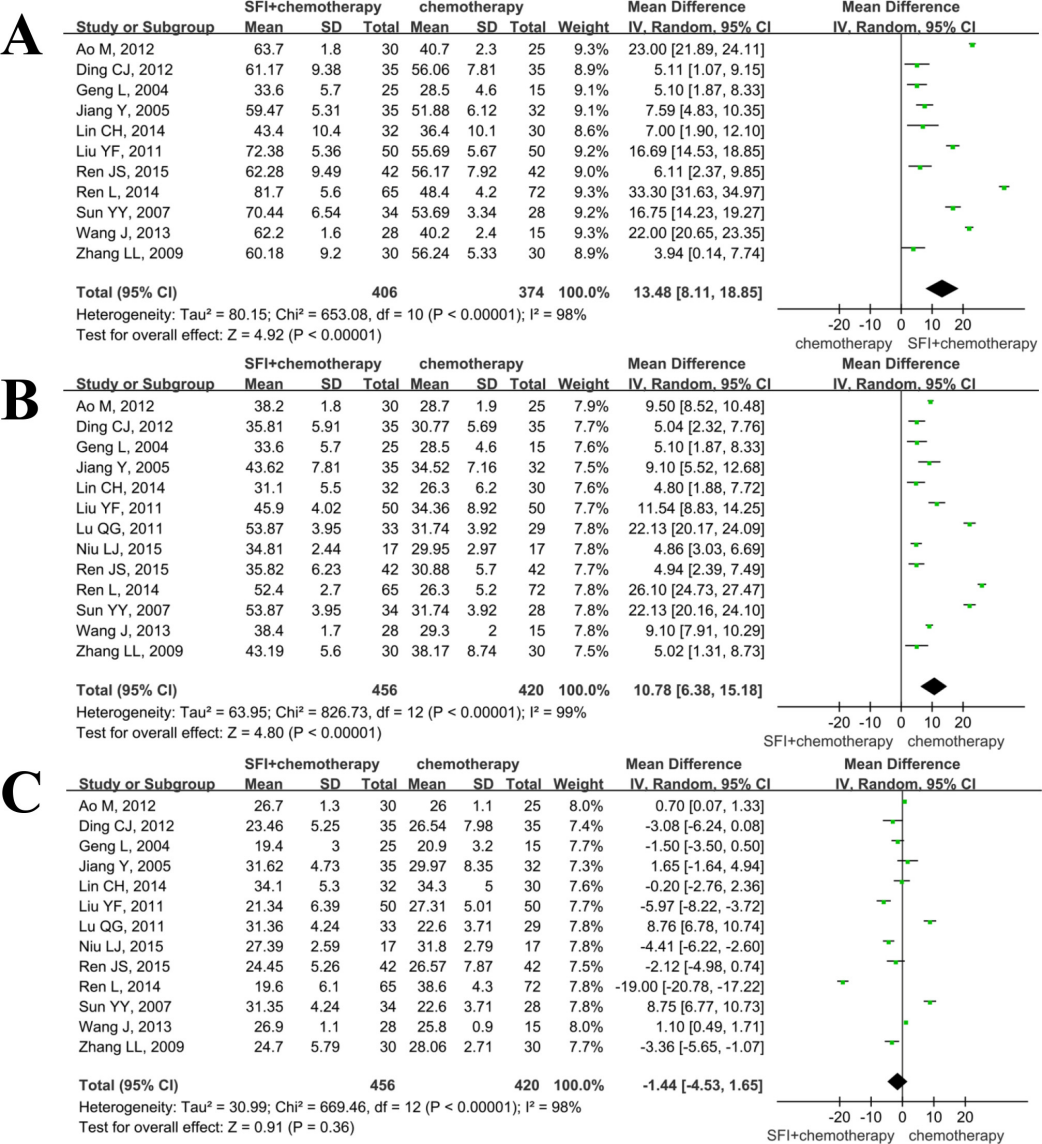
Meta analysis of SFI on the improvement of cellular immune indicators when combined with chemotherapy in patients with advanced NSCLC. A: percentage of CD_3_^+^ was increased when using SFI; B: percentage of CD_4_^+^ was increased when using SFI; C: percentage of CD_8_^+^ was increased when using SFI.

Eleven studies[10, 11, 14–17, 19, 20, 24–26] included in this systematic analysis provided the data on percentage of CD_3_^+^ after treatment. As there was statistical heterogeneity(I^2^ = 98%, *p*<0.01), the random effect model was used in this meta analysis. The results showed that the SMD was 13.48(95%CI: 8.11–18.85; p<0.01), indicating that application of SFI could increase the percentage of total lymphocytes(Fig 3A).

Thirteen articles[10, 11, 14–21, 24–26] reported percentages of helper T lymphocytes(CD_4_^+^) at the end of the treatment. These data were not found to be homogeneous(I^2^>50%), applying the random effect model in this meta-analysis. As shown in Fig 3B, SFI had an advantage of increasing the percentage of helper T lymphocytes compared to control group(SMD = 10.78; 95%CI, 6.38–15.18; *p*<0.01).

There were 13 trials[10, 11, 14–21, 24–26] included in the meta analysis of improvement of percentages of cytotoxic T lymphocytes (CD_8_^+^). Significant heterogeneity was detected among data from the included studies(I^2^>50%). As illustrated by Fig 3C, the results illustrated that the percentage of CD_8_^+^ cells was similar between SFI combination group and control group(SMD = -1.44; 95%CI, -4.53–1.65; *p* = 0.36).

**Fig 4:**
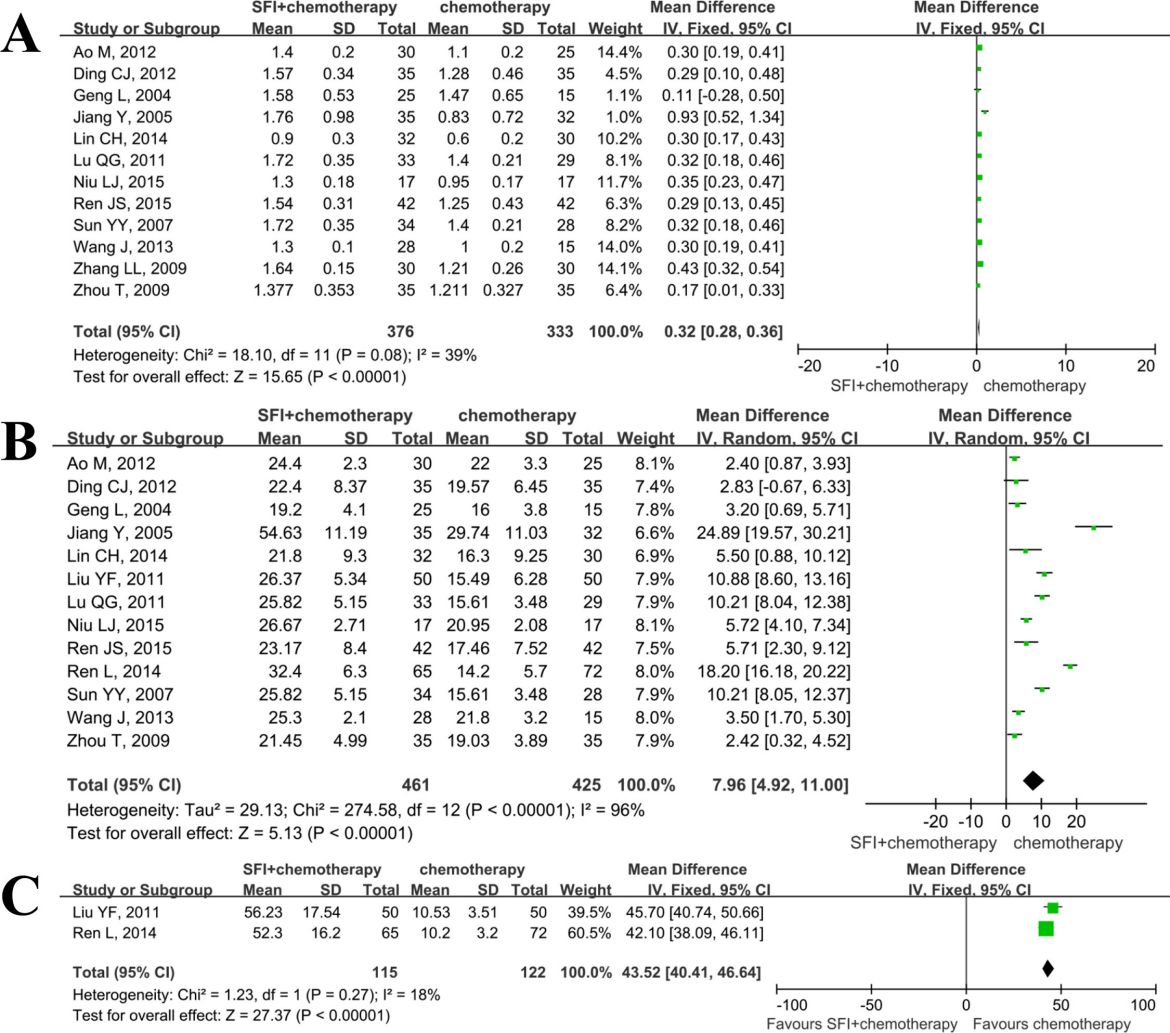
Meta analysis of SFI on other aspects of immune function when combined with chemotherapy in patients with advanced NSCLC. A: ratio of CD_4_^+^/CD_8_^+^ was increased when using SFI; B: percentage of NK cells was increased when using SFI; C: lymphocyte transformation rate was increased when using SFI.

12 studies[10, 11, 14, 15, 17–21, 23, 25, 26] reported the data on CD_4_^+^/CD_8_^+^ increased in both groups. There was no statistically significant heterogeneity among these studies(I^2^ = 39%), so the fixed effect model was used(Fig 4A). The results showed that there was significant difference in increased extent of CD_4_^+^/CD_8_^+^ between the combination group and the chemotherapy group (SMD = 0.32; 95%: 0.28–0.36; *p*<0.01).

Thirteen articles[10, 11, 14–21, 24–26] provided data on changes of percentages of NK after treatment. The I^2^ test found that the data was not homogeneous(I^2^>50%), justifying the random effect model in this meta-analysis. As shown in Fig 4B, SFI group exhibited significant improvement of percentage of NK cells, compared with chemotherapy group(SMD = 7.96; 95%CI, 4.92–11.00; *p*<0.01). The lymphocyte transformation rate was also significantly increased when using SFI(Fig 4C).

**Fig 5:**
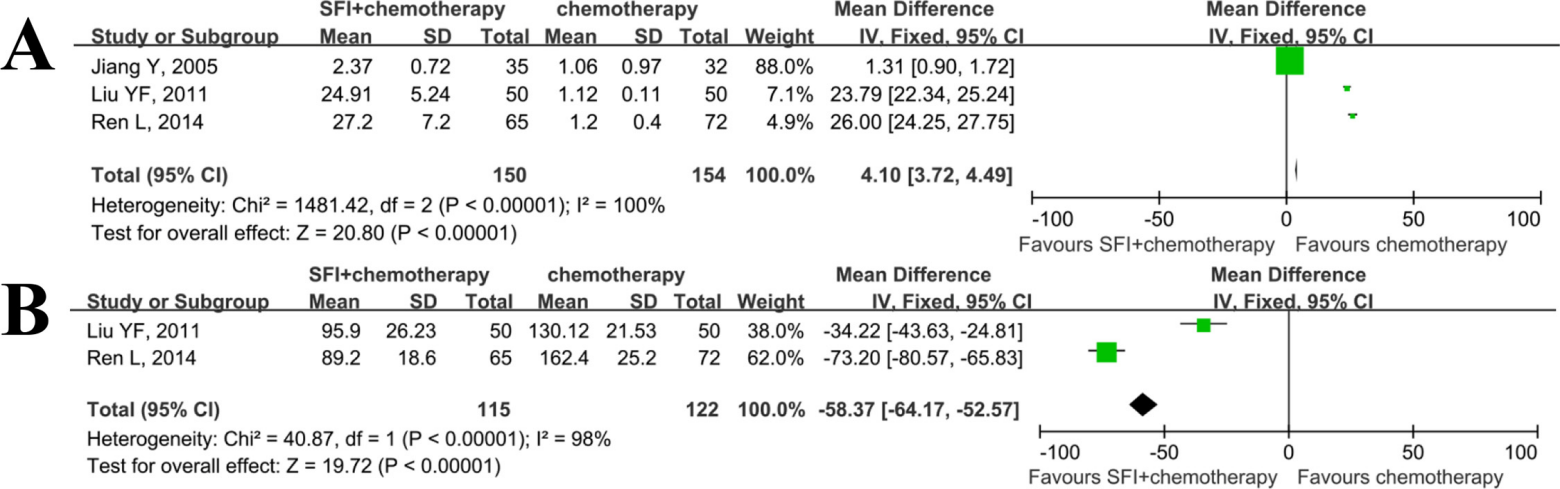
Meta analysis of SFI on concentration of IL-2 and TNF-alpha when combined with chemotherapy in patients with advanced NSCLC. A: level of IL-2 was up-regulated when using SFI; B: level of TNF-alpha was down-regulated when using SFI.

Few studies[16, 24, 25] also evaluated the effect of SFI on the levels of IL-2(Fig 5A) and TNF-alpha(Fig 5B). The SMD for IL-2 and TNF-alpha were 17.02(95%CI: -1.41–35.46; p = 0.07) and -53.82(95%CI: -92.02–-15.62; p = 0.006), respectively.

### Detoxication of SFI combined with chemotherapy

**Fig 6:**
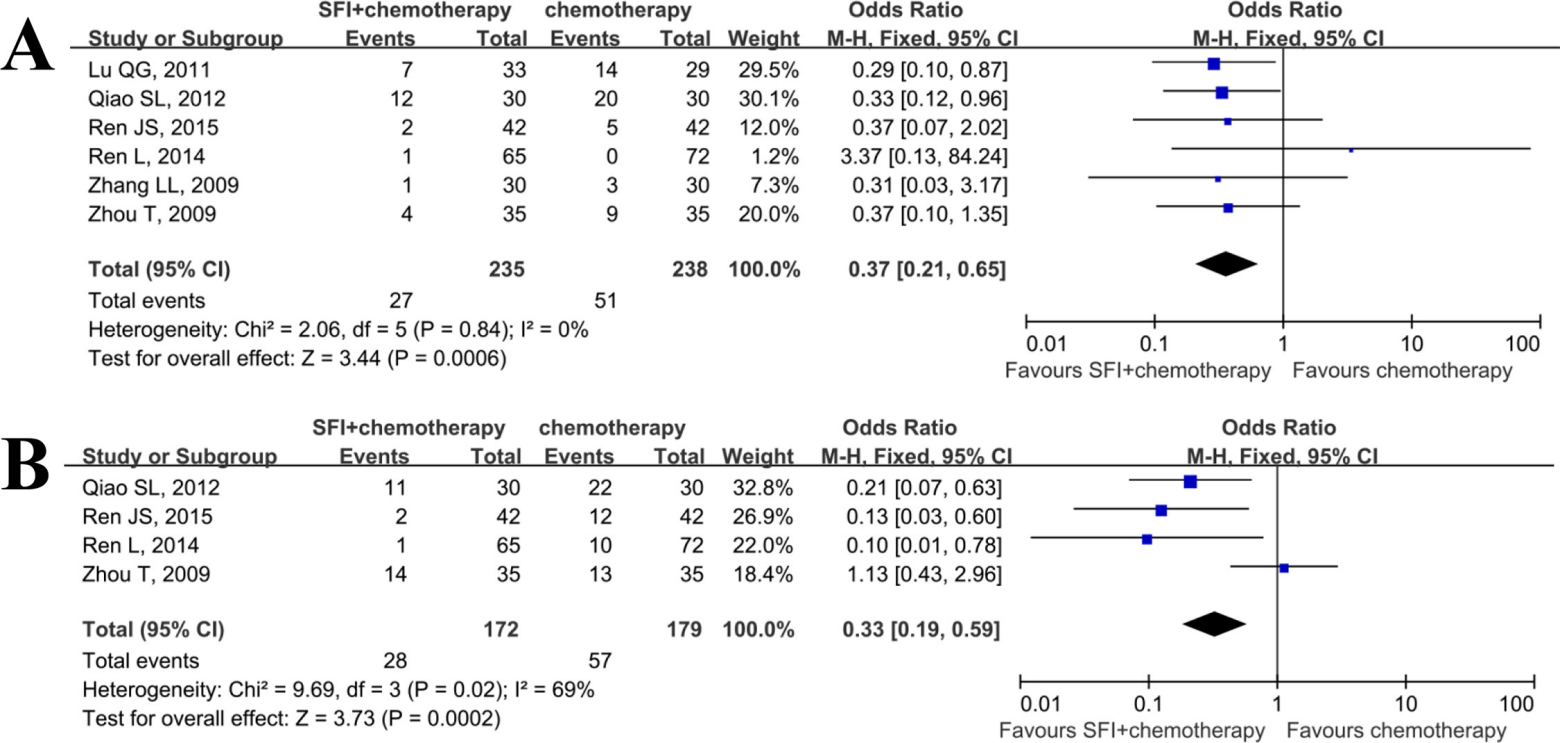
Meta analysis of SFI on adverse events when combined with chemotherapy in patients with advanced NSCLC. A: granulopenia; B: nausea and vomiting.

As eligible data on toxicity of treatment was extracted from only six trials[10, 16, 19, 21–23], making it difficult to confirm the capability of detoxication of SFI when combined with chemotherapy. Nevertheless, we performed this meta analysis to assess whether SFI could reduce the incidence of severe granulopenia and nausea and vomiting, and the results were positive(Fig 6). The OR for granulopenia(Fig 6A) was 0.37(95%CI: 0.21–0.65; p<0.01), while the OR for nausea and vomiting(Fig 6B) was 0.33(95%CI: 0.19–0.59; p<0.01).

### Improvement of QOL of SFI

**Fig 7:**
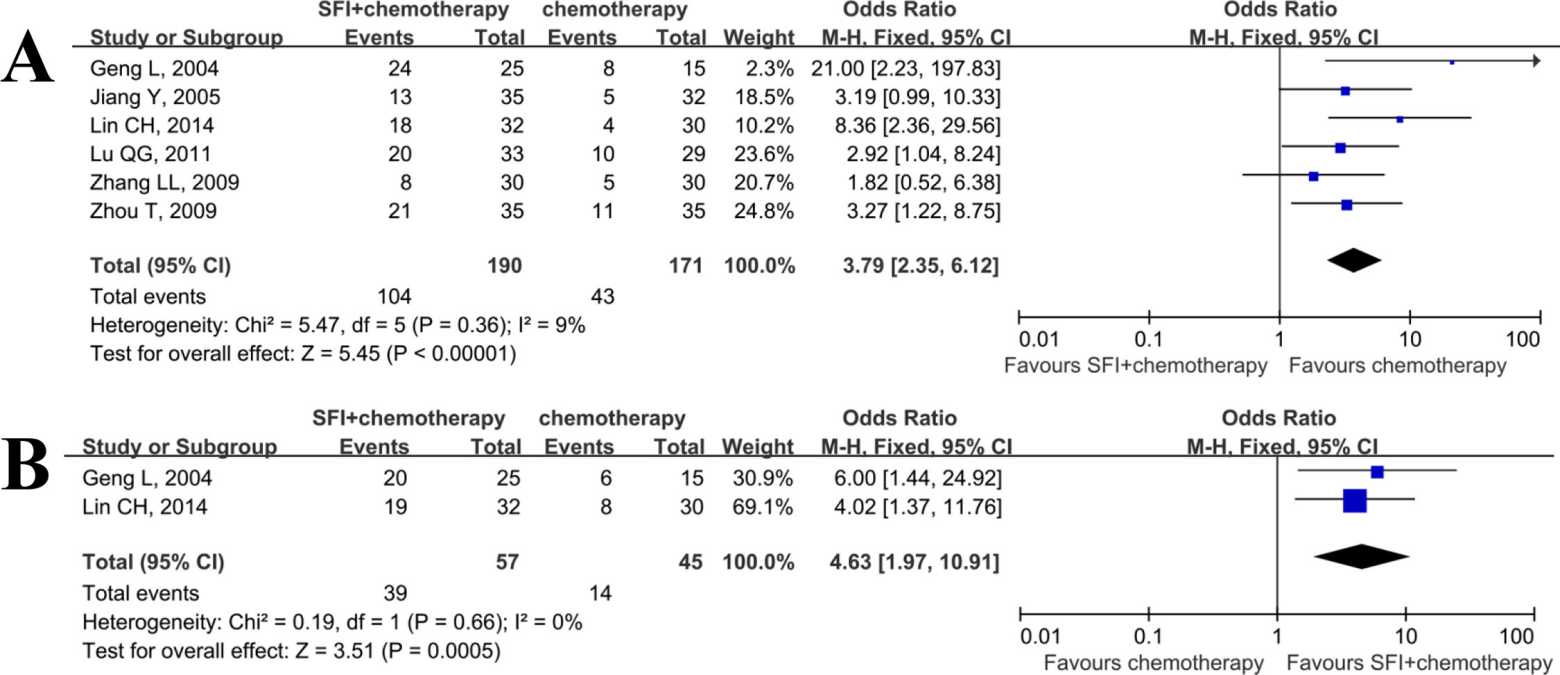
Meta analysis of SFI on quality of life and weight gain when combined with chemotherapy in patients with advanced NSCLC. A: quality of life; B: weight gain.

The number of patients with improved KPS were provided by 6 studies [14, 17, 19, 21, 23, 25]. It proved to be homogeneous, so the fixed effect model was used in this meta-analysis. The results exhibited a statistically significant better improvement of KPS when SFI combined with chemotherapy group was compared with chemotherapy group (RR 2.08; 95% CI: 1.57–2.74; P<0.01; Fig 7A), indicating that addition of SFI during chemotherapy could result in better quality of life.

We also assessed the effect of SFI on the weight gain in patients with advanced NSCLC. However, only two studies were able to provide the data on weight gain. The result revealed that patients received SFI treatment may slightly increase their body weight than control group(RR 2.12; 95% CI: 1.33–3.37; P = 0.002; Fig 7B).

### Analysis of publication bias

**Fig 8:**
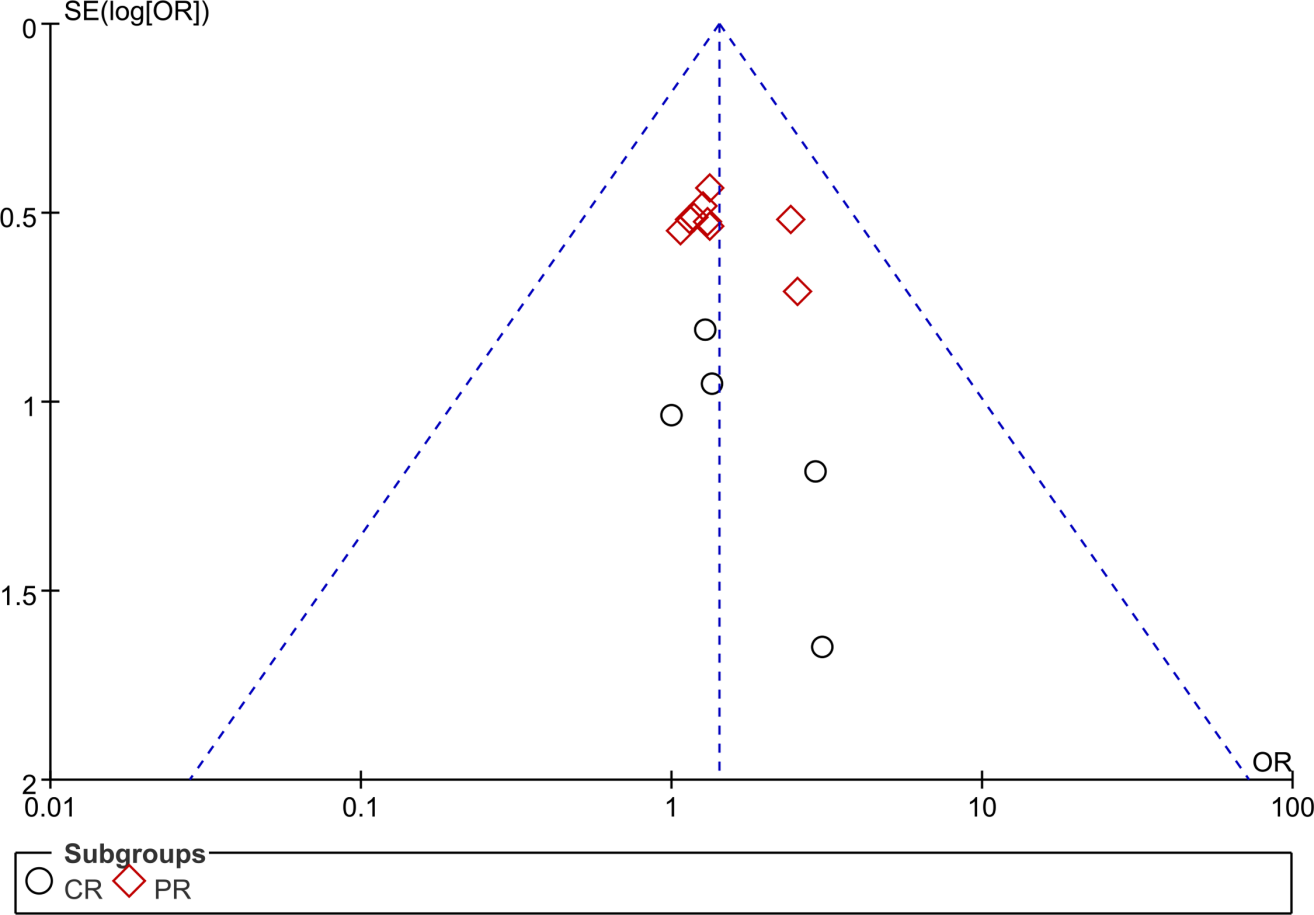
The funnel plot for assessing publication bias of ORR.

As the most included studies were reporting the immunity, we chose this data parameter to perform the analysis of publication bias. The funnel plot generated form studies reporting efficacy was asymmetric, suggesting that significant publication bias existed in the included trials(Fig 8). Furthermore, Egger's test proved the presence of obvious bias of publication(p<0.05).

## Discussion

In recent years, with the recognition of evidence-based medicine, systematic review and meta analysis have been widely accepted and used to guide the clinical practice, ensuring that interventions are based on the most reliable conclusions[27]. The significant advantage of meta analysis is that its findings are based on statistical synthesis of RCTs on the same question. We performed this meta analysis to assess the impact of SFI on efficacy, quality of life and immunity of advanced NSCLC patients when combined with chemotherapy. A total of 15 studies reporting SFI + chemotherapy versus chemotherapy in patients with advanced NSCLC were included. And the results showed that improvements in tumor response rate, cellular immune function, QOL, and reduction in adverse events such as severe grade of nausea and vomiting, and granulopenia were observed in SFI combination group. In addition, SFI also exhibited the ability of not only keeping, but also increasing weight, when compared to control group. Though the related studies and clinical results are mainly limited in China, and difficult to be found through online academic databases in western countries, the positive effect of SFI should not be ignored and underestimated. The results of our meta analysis seemed to indicate that SFI may exert its anti-tumor effect via enhancing the immune function of cancer patients, which is in accordance with the traditional role of SFI on "Qi" in China. This comprehensive review provided a more precious conclusion with regards to SFI.

SFI is mainly made from Codonopsis pilosula and Astragali, which are two traditional Chinese medicine and used to modulate the immune function of patients with severe illnesses, such as cancers[28]. Results of several clinical studies performed in China have demonstrated that improvements of percentages of total T lymphocytes, helper T lymphocytes, cytotoxic T lymphocytes, CD_4_^+^/ CD_8_^+^, and natural killer cells, which are indicators of cellular immune function, are observed in NSCLC patients[11, 15, 16, 20, 24, 28]. The concentration of IL-2 and TNF-alpha are also differently regulated, which may result in enhancing the ability of tumor controlling for cancer patients[29, 30]. In this meta analysis, we collected data relevant to immunity and pooled them together to find out whether SFI did have an effect on the immune function of advanced NSCLC patients. The results showed that the percentages of total T lymphocytes, helper T lymphocytes, cytotoxic T lymphocytes, and natural killer cells SFI were improved, respectively. With regards to the ORR, it was also found that the pooled ORR in SFI combination group was higher than that in the chemotherapy alone group, however, no significant difference was found. This was partly in accordance with a meta analysis of SFI treatment for NSCLC patients published in 2010[31]. In that meta analysis, Ju Dong, et al. included 29 studies, and the results exhibited that significantly higher tumor response (RR, 1.19; 95% CI, 1.07 to 1.32; P = 0.001) was found when the SFI combination group was compared with chemotherapy group[31]. Different inclusion criteria may be responsible for the slightly inconsistent findings between these two meta analysis. Studies reporting both clinical efficacy and cellular immunity parameters were included in this study and the included studies of Ju Dong et al. were mainly about clinical efficacy. Nevertheless, patients received SFI during chemotherapy did show better response, quality of life, and less adverse events than chemotherapy alone. The underlying mechanisms of anti-tumor effect of SFI are now being investigated using modern technology and methods.

The application of TCMs in treating malignant diseases have been lasting for decades. In recent years, several meta-analysis about the efficacy of traditional Chinese medicine in combination with chemotherapy or radiotherapy have been reported[32–37]. The reviews performed by Chen, et al.[32–34] assessed the role of TCM in treating NSCLC when combined with chemotherapy, and they concluded that TCM could improve QOL, prolong survival rate, enhance immediate tumor response and reduce toxicity when combined with chemotherapy, either in the form of oral or injection. Astragalus is one of the main components of SFI, and it has been proven to be capable of enhancing immunologic function. McCulloCh, et al[35] performed a systemic review to evaluate the efficacy of astragalus-based Chinese drugs in curing advanced NSCLC, and the results suggested that astragalus-based medicine may increase effectiveness when combined with chemotherapy, which is in consistent with the present study, although SFI was not included in their study. Moreover, Jean et al.[36] further reported that herbal treatment could benefit 4751 NSCLC patients from 65 RCTs with significantly improved survival at 12, 24 and 36 months. However, the conclusion was limited due to the poor quality of reporting of RCTs. Besides chemotherapy, SFI could also improve the clinical efficacy of radiation(RR = 1.27, 95% CI: 1.13–1.43) and reduce radiation related adverse events, such as radiation related pneumonia(RR = 0.41, 95% CI: 0.26–0.46), esophagitis(RR = 0.46, 95% CI: 0.37–0.59), and myelosuppression (RR = 0.44, 95% CI: 0.36–0.53) in the NSCLC patients[37]. These studies suggest that TCM, especially SFI, could improve the efficacy and QOL, decrease adverse events of chemotherapy or radiation in NSCLC patients, which is also indicated by our review. However, the underlying mechanisms of these effects are not well illustrated and should be explored and clarified using modern research technologies in urgency.

Several limitations are existed in this review and meta analysis. First, though we defined current primary online databases, such as Pubmed, EMBASE, and the cochrane library, as the main sources of eligible studies with the language restriction of both English and Chinese, most of the included trials relating to SFI+ chemotherapy versus chemotherapy for advanced NSCLC patients were found in Chinese, indicating publication bias may exist across studies. Secondly, only few studies reported information of allocation and blinding, and this could be regarded as there were high risks of selection and performance bias, lowering the overall quality of included studies. Thirdly, the number of included patients, chemotherapy regimen, and duration of SFI treatment were inconsistence between studies, increasing the risk of heterogeneity. In addition, we only included studies containing data of both efficacy and immunity, this could lead to a higher risk of selection bias. Despite these limitations, this study still provides mild evidence to support that the application of SFI for immunocompromised cancer patients receiving chemotherapy could be favorable. And it needed to be confirmed further.

## Conclusion

The application of traditional Chinese medicine including SFI during chemotherapy for advanced cancer patients have been widely practiced for years in China[38]. In recent years, it showed that the addition of SFI to chemotherapy could improve the objective response rate and immune function for advanced NSCLC patients. Our meta analysis also demonstrated these findings. Due to the difficulty of personalized treatment of traditional Chinese medicine for advanced cancer patients, it is nearly impossible to conduct large number, double-blind, randomized controlled clinical trials, making the findings of this study hard to be further confirmed. Fortunately, by using modern medical technology, increasing investigators have focused their attentions on the underlying mechanisms of how SFI could improve the immune functions of cancer patients. Their efforts will support our findings with a different perspective. Overall, although the quality of the included studies are relatively insufficient, the results could still be useful to provide clinical evidence for the advantage of SFI treatment.

## Supporting Information

S1 Table The 12 full-text excluded articles with reasons. (DOCX)

S1 File The PRISMA 2009 Checklist. (DOC)
