# Supplementary material for: The Effect of ShenQi FuZheng Injection in Combination with Chemotherapy versus Chemotherapy Alone on the Improvement of Efficacy and Immune Function in Patients with Advanced Non-Small Cell Lung Cancer: A Meta-Analysis
Source: PLoS One. 2016 Mar 25;11(3):e0152270. doi: 10.1371/journal.pone.0152270 (PMC4807845; doi:10.1371/journal.pone.0152270)
Supplement: S1 Table — (DOCX) [file pone.0152270.s001.docx]

S1 Table The 12 full-text excluded articles with reasons

| Excluded articles | Reasons |
| --- | --- |
| Dong J, et al.[[1](#_ENREF_1)] | review |
| Li J, et al.[[2](#_ENREF_2)] | review |
| Qi F, et al.[[3](#_ENREF_3)] | one arm study |
| She J, et al.[[4](#_ENREF_4)] | review |
| Wang J, et al. [[5](#_ENREF_5)] | animal study |
| Yin JY, et al.[[6](#_ENREF_6)] | study without reporting immune function |
| Wang J, et al.[[7](#_ENREF_7)] | study without reporting immune function |
| Jiang H, et al.[[8](#_ENREF_8)] | review |
| Li SG, et al.[[9](#_ENREF_9)] | review |
| Chen, S, et al.[[10](#_ENREF_10)] | review |
| Jung-Woo Lee, et al.[[11](#_ENREF_11)] | review |
| Li X, et al.[[12](#_ENREF_12)] | review |

References

1. Dong J, Su SY, Wang MY, Zhan Z. Shenqi fuzheng, an injection concocted from Chinese medicinal herbs, combined with platinum-based chemotherapy for advanced non-small cell lung cancer: a systematic review. Journal of experimental & clinical cancer research : CR. 2010;29:137. doi: 10.1186/1756-9966-29-137. PubMed PMID: 20969765; PubMed Central PMCID: PMC2972256.

2. Li J, Wang JC, Ma B, Gao W, Chen P, Sun R, et al. Shenqi Fuzheng Injection for advanced gastric cancer: a systematic review of randomized controlled trials. Chinese journal of integrative medicine. 2015;21(1):71-9. doi: 10.1007/s11655-014-1768-8. PubMed PMID: 25246138.

3. Qi F, Zhao L, Zhou A, Zhang B, Li A, Wang Z, et al. The advantages of using traditional Chinese medicine as an adjunctive therapy in the whole course of cancer treatment instead of only terminal stage of cancer. Bioscience trends. 2015;9(1):16-34. doi: 10.5582/bst.2015.01019. PubMed PMID: 25787906.

4. She J, Yang P, Hong Q, Bai C. Lung cancer in China: challenges and interventions. Chest. 2013;143(4):1117-26. doi: 10.1378/chest.11-2948. PubMed PMID: 23546484.

5. Wang J, Tong X, Li P, Cao H, Su W. Immuno-enhancement effects of Shenqi Fuzheng Injection on cyclophosphamide-induced immunosuppression in Balb/c mice. Journal of ethnopharmacology. 2012;139(3):788-95. doi: 10.1016/j.jep.2011.12.019. PubMed PMID: 22212503.

6. Yin JY, Huang Q, Zhao YC, Zhou HH, Liu ZQ. Meta-analysis on pharmacogenetics of platinum-based chemotherapy in non small cell lung cancer (NSCLC) patients. PloS one. 2012;7(6):e38150. doi: 10.1371/journal.pone.0038150. PubMed PMID: 22761669; PubMed Central PMCID: PMC3383686.

7. Wang J, Tong X, Li P, Liu M, Peng W, Cao H, et al. Bioactive components on immuno-enhancement effects in the traditional Chinese medicine Shenqi Fuzheng Injection based on relevance analysis between chemical HPLC fingerprints and in vivo biological effects. Journal of ethnopharmacology. 2014;155(1):405-15. doi: 10.1016/j.jep.2014.05.038. PubMed PMID: 24950446.

8. Jiang H, Zhang H, Hu X, Ma J. A meta-analysis of Shenqi Fuzheng combined with radiation in the treatment of nonsmall cell lung cancer. Journal of cancer research and therapeutics. 2015;11 Suppl 1:C101-3. doi: 10.4103/0973-1482.163855. PubMed PMID: 26323903.

9. Li SG, Chen HY, Ou-Yang CS, Wang XX, Yang ZJ, Tong Y, et al. The efficacy of Chinese herbal medicine as an adjunctive therapy for advanced non-small cell lung cancer: a systematic review and meta-analysis. PloS one. 2013;8(2):e57604. doi: 10.1371/journal.pone.0057604. PubMed PMID: 23469033; PubMed Central PMCID: PMC3585199.

10. Chen S, Flower A, Ritchie A, Liu J, Molassiotis A, Yu H, et al. Oral Chinese herbal medicine (CHM) as an adjuvant treatment during chemotherapy for non-small cell lung cancer: A systematic review. Lung cancer. 2010;68(2):137-45. doi: 10.1016/j.lungcan.2009.11.008. PubMed PMID: 20015572.

11. Lee J-W, Kim W, Min B-I, Baek SK, Cho S-H. Traditional herbal medicine as an adjuvant treatment for non-small-cell lung cancer: A systematic review and meta-analysis. European Journal of Integrative Medicine. 2015;7(6):577-85. doi: http://dx.doi.org/10.1016/j.eujim.2015.08.005.

12. Li X, Yang G, Li X, Zhang Y, Yang J, Chang J, et al. Traditional Chinese medicine in cancer care: a review of controlled clinical studies published in chinese. PloS one. 2013;8(4):e60338. doi: 10.1371/journal.pone.0060338. PubMed PMID: 23560092; PubMed Central PMCID: PMC3616129.
